# Modality-Dependent Brain Activation Changes Induced by Acquiring a Second Language Abroad

**DOI:** 10.3389/fnbeh.2021.631957

**Published:** 2021-03-26

**Authors:** Kuniyoshi L. Sakai, Tatsuro Kuwamoto, Satoma Yagi, Kyohei Matsuya

**Affiliations:** Department of Basic Science, Graduate School of Arts and Sciences, The University of Tokyo, Tokyo, Japan

**Keywords:** language acquisition, syntax, fMRI, hippocampus, learning and memory (neurosciences)

## Abstract

The dynamic nature of cortical activation changes during language acquisition, including second-language learning, has not been fully elucidated. In this study, we administered two sets of reading and listening tests (Pre and Post) to participants who had begun to learn Japanese abroad. The two sets were separated by an interval of about 2 months of Japanese language training. We compared the results of longitudinal functional MRI experiments between the two time-points and obtained the following major findings. First, the left-dominant language areas, as well as bilateral visual and auditory areas, were activated, demonstrating the synergistic effects of multiple modalities. There was also significant activation in the bilateral hippocampi, indicating the expected involvement of memory-related processes. Second, consistent with the behavioral improvements from Pre to Post, the brain activations *decreased* in the left inferior and middle frontal gyri during the listening tests, as well as in the visual areas (the bilateral inferior and superior parietal lobules, and left inferior and middle occipital gyri) during the reading tests, while activations in the right superior and middle temporal gyri *increased* during the listening tests. These modality-dependent activation changes could not be explained by domain-general cognitive factors, such as habituation or familiarization, because we used completely different test sets for Pre and Post. Third, the posterior hippocampus showed a main effect of the hemisphere, whereas the anterior hippocampus showed a significant main effect of the event (i.e., specific to first listening events), reflecting initial encoding of auditory information alone. In summary, activation changes from Pre to Post indicate functional changes in modality-dependent networks over a short period of staying abroad, which would enable effective acquisition of a second language.

## Introduction

Recent advances in human neuroimaging studies have revealed both anatomical and functional changes during second language (L2) acquisition (Chee et al., 2001; Reiterer et al., 2009; Schlegel et al., 2012; Li et al., 2014), which occur in the language-related regions required for first or native languages (L1), and possibly in other regions as well. By using diffusion magnetic resonance imaging (diffusion MRI), we have recently shown that the structural measure (fractional anisotropy) of the left arcuate fasciculus (i.e., dorsal pathway) connecting the left inferior frontal gyrus (IFG) and other language-related regions was significantly correlated with performance on a syntactic task in high-school students who had studied English as an L2 at school (Yamamoto and Sakai, 2016), and this correlation was clearly dissociated from developmental changes (Yamamoto and Sakai, 2017). Another MRI study performed during 16 weeks of vocabulary training in L2 for university students showed a volume increase in the *right* IFG, as well as a connectivity change in the right hemisphere (Hosoda et al., 2013). Although the issue of hemispheric dominance remains, such anatomical changes during L2 acquisition likely affect the functions of those regions as well.

Each of the language-related regions in the left hemisphere has a specific function irrespective of modalities (i.e., audition or vision) or input/output. For example, the dorsal and ventral regions of the left IFG are specialized in syntax and sentence comprehension, respectively, whereas the left superior temporal regions and angular/supramarginal gyri subserve phonological and lexico-semantic processing, respectively (Sakai, 2005). By using functional MRI (fMRI), we previously compared two groups of high-school students, whose ages of acquisition (AOA) in L2 were 6 years apart, and demonstrated that activations of the left IFG correlated positively with the accuracy of a syntactic task for the *late* learners (mean AOA: 12.6), whereas activations of the left ventral IFG correlated negatively with the accuracy for the *early* learners (mean AOA: 5.6; Sakai et al., 2009). Although some fMRI studies support the idea that AOA affects cortical activations, such that the left IFG activation for grammatical processing in L2 is greater than that in L1 (Wartenburger et al., 2003), other fMRI studies have concluded that the degree of exposure to language affects the left IFG activation, even if the AOA is matched (Perani et al., 2003). To resolve these conflicting claims, we have proposed that the cortical activations may initially increase upon onset of acquisition, then remain at the increased level for some time, and finally fall during the consolidation of linguistic competence (see Figure 3E in Sakai, 2005). If this general law applies to L2 in general, then the language-related regions may show higher, lower, or comparable activation, depending on which developmental phases or aspects are compared. It is also probable that the time course of L2 acquisition depends on the specific linguistic function we focus on.

**Figure 1:**
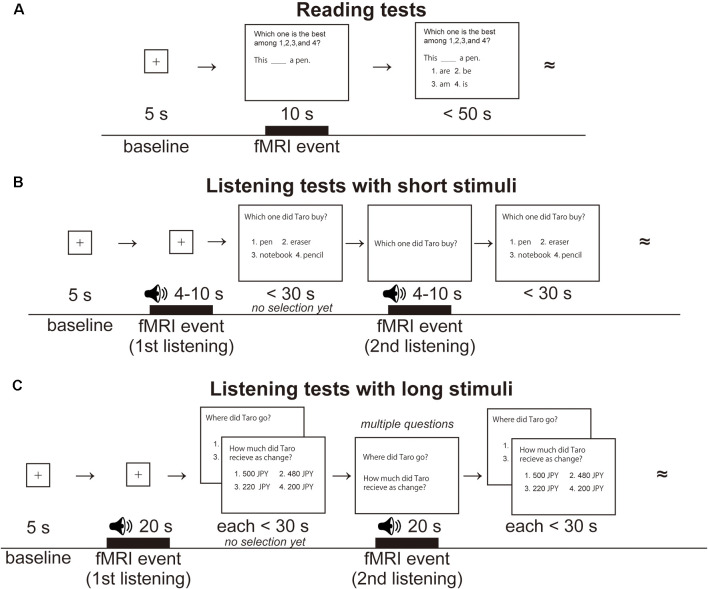
Examples of reading and listening tests for learning Japanese as a second language. **(A)** A single trial of the reading tests. Following the presentation of a fixation point for 5 s (baseline for brain activation), a test sentence was visually presented for 10 s (an fMRI event of *reading*), and then four choices were additionally presented as possible replacements for the underlined portion of the sentence. The participants were required to select one of the choices within 50 s. The double tilde symbols denote continuation of similar trials in that run. Visual stimuli were presented in Japanese, but English equivalents are shown here. **(B)** A single trial of the listening tests with short stimuli. The same speech stimulus was presented twice, for 4–10 s each time (fMRI events labeled *first listening* and *second listening*). After listening to the first stimulus, the participants were visually presented with a question sentence and four possible answers and were given 30 s to proceed without choosing an answer. After listening to the second stimulus, the participants finally chose one of the four answers by pressing a button. Both auditory and visual stimuli were presented in Japanese, but English equivalents are shown here. **(C)** A single trial of the listening tests with long stimuli. The same speech stimulus was presented twice, for 20 s each time (fMRI events labeled *first listening* and *second listening*), together with multiple questions. English equivalents are shown here.

It is also likely that modality-specific regions, i.e., auditory and visual areas, would also show activation changes during L2 training for listening and reading abilities. In our previous study on newly learned Hangul letters with speech sounds, we showed a positive correlation between accuracy improvements and activation *increases* in the left posterior inferior temporal gyrus (Hashimoto and Sakai, 2004), indicating that activation changes occur even within two consecutive *days* during the initial stage of learning. It would be interesting to examine whether cortical activations continue to increase or rather begin to decrease after several *months* of exposure to newly acquired speech sounds and letters in L2, still at the early stage of training. In our present longitudinal experiments, we conducted reading and listening tests (see Figure 1) before and after about 2 months of L2 training for each participant, and examined the differences in brain activation between the two time-points. The reading and listening tests in L2 were similar to those used in a classroom, where auditory stimuli are usually presented twice: first listening for general comprehension, and second listening for more selective focusing on specific questions. The reading and listening tests differed not only in their modalities but in their question contents and presentations (e.g., visual material can be read repeatedly). However, both syntactic and semantic processes were crucially involved in both types of tests, and we should bear in mind that modality-dependent effects examined here may contain such subsidiary processes as well.

During the initial L2 training, language-related regions, as well as auditory and visual areas, are expected to be crucially involved. Because they are the most critical regions of syntactic processing, we predict that the left inferior and middle frontal gyri (L. IFG/MFG) will exhibit activation changes. The L. IFG/MFG have been proposed as grammar centers (Sakai, 2005), and we have recently clarified that their right-side homologs, the right IFG/MFG (R. IFG/MFG), play a supportive role as a part of the syntax-related network I (Kinno et al., 2014; Tanaka et al., 2020). The auditory areas include the bilateral superior and middle temporal gyri (STG/MTG), and the visual areas may include at least the bilateral inferior and superior parietal lobules (IPL/SPL), fusiform gyrus (FG), and inferior and middle occipital gyri (IOG/MOG). Another candidate region involved in L2 acquisition would be the hippocampus due to its role in processing episodic memory (Zeidman and Maguire, 2016), although its role in the process of language acquisition has not been fully elucidated. While an fMRI study suggested that an increased proficiency level of an artificial language was associated with decreased activity in the left posterior hippocampus (Opitz and Friederici, 2003), the left posterior hippocampus is selectively activated for memory retrieval of word associations (Prince et al., 2005). The objectives of the present study were to clarify how each of these cortical regions and hippocampi showed activations depending on different modalities and time-points during L2 acquisition.

## Materials and Methods

### Participants

We recruited 18 participants, who had learned Japanese through EF (EF Education First, Switzerland) courses for the first time at the EF Tokyo campus in Shibuya. During their 6-12 months stay abroad in Japan, the participants were exposed to Japanese in various rich social environments for at least 3 h each day. To focus on activation changes at the early stage of training in a second language, one participant who had studied Japanese for 6 years before the stay was dropped. The other participants had no history of learning Japanese before their stay in Japan. We assessed handedness according to the Edinburgh inventory (Oldfield, 1971), and dropped two participants whose laterality quotients showed left-handedness. The remaining 15 participants (10 males and 5 females) were 21 ± 3.5 years old [mean ± standard deviation (SD)], and showed right-handedness (laterality quotients: 79 ± 26). Their L1s were German (7), Norwegian (4), Spanish (2), French (1), and Dutch (1); they had also learned English as L2 mostly at school.

All experiments were performed as per relevant guidelines and regulations, including the Declaration of Helsinki, and the Singapore Statement on Research Integrity. All participants provided their written informed consent to participate in this study after the nature and possible consequences of the study were explained. Approval for these experiments was obtained from the institutional review board of the University of Tokyo, Komaba Campus.

### Stimuli and Tasks

We designed two sets of tests (A and B) from EF placement tests in Japanese, each of which consisted of 25 reading and five listening tests, including questions about syntactic structures and sentence comprehension. Auditory stimuli were speech sounds taken from EF placement tests in Japanese. During the EF course of Japanese for beginners, different sets of tests (*Pre* and *Post*) were administered 40–100 days apart (61 ± 18 days), where the Pre sets followed 2 or 3 months of initial courses at the beginning of their stay in the L2 environment. The order of sets A and B was counterbalanced across participants.

For the reading tests, participants read a short sentence with a missing phrase or particle, and chose one from four suggested replacements. All visual stimuli were presented in white against a dark background. For fixation, a small red cross was shown at the center of the screen to initiate eye movements from the same fixed position, and the participants were instructed to return their eyes to this position. During the scans, the participants wore an eyeglass-like MRI-compatible display (resolution = 800 × 600, framerate = 60 fps; VisuaStim Digital, Resonance Technology Inc., Northridge, CA, USA). For weak-sighted participants, correction lenses were inserted in front of the display.

For each reading test, we presented a fixation point for 5 s, which was used as a baseline for the fMRI analyses (Figure 1A). A test sentence with a missing phrase or particle was visually presented for 10 s (an fMRI event of *reading*), and then four choices were presented to fill in the blank. Participants read the stimuli and responded within 50 s while the sentence was presented, which was the maximum time set for beginners. A set of reading tests were presented in three different writing systems, corresponding to the three steps of learning to read in Japanese: five sentences written in the Roman alphabet of Japanese sounds (easiest notation for Europeans), 15 sentences in *kana* (a phonetic alphabet in Japanese, including both *hiragana* and *katakana*), and five sentences in *kana* and *kanji* (with Chinese characters, in a normal writing style). The leading questions (e.g., “Which one is the best among 1, 2, 3, and 4?”) and choices were presented in *kana* alone. For each participant, we tested five scanning runs, where each run included five reading tests.

For the listening tests, not only *short* stimuli of a brief Japanese sentence for 4–10 s (Figure 1B), but *long* stimuli of multiple sentences for 20 s were administered to examine different aspects of listening abilities (Figure 1C). The former involved a basic knowledge about sentence construction and comprehension of a single sentence, while the latter required further syntactic and contextual understanding of connected sentences; we combined both aspects in the subsequent analyses. Only one straightforward question was assigned to each short stimulus, whereas two or three questions were associated with each long stimulus. Test set A included three short stimuli with one question each, and a long stimulus with two questions; test set B included two short stimuli with one question each, and a long stimulus with three questions. For each participant, we tested a scanning run with five listening tests (i.e., five questions).

For each listening test with a short stimulus, we presented a fixation point as a baseline, and then speech sounds were presented for 4–10 s (an fMRI event of *first listening*). The first listening event was the initial pure exposure to speech stimuli without performing a task or seeing any visual material. Next, a question was visually presented in *kana* with four choices to let the participants know what would be asked on the auditory stimuli; the participants were given up to 30 s to read this material, and indicated their readiness to proceed, without choosing an answer, by pressing *any* of the four buttons. The same speech sounds were presented again while the same question set (but without choices) was visually presented (an fMRI event of *second listening*), which simulated a listening test with questions on the display, like a TOEFL (Test of English as a Foreign Language) listening test; this event thus involved multimodal integration. After listening to the second stimulus, the participants finally chose one of the four answers by pressing an appropriate button. Concerning each listening test with a long stimulus (20 s), the differences from the listening test with short stimuli were the duration of fMRI events and the presence of multiple questions, as stated above.

During the scans, the participants wore an MRI-compatible headphone, VisuaStim Digital (Resonance Technology Inc., Northridge, CA, USA), a pair of earmuffs (3M Peltor, St. Paul, MN, USA), and a pair of earplugs (Earasers, Persona Medical, Casselberry, FL, USA) to reduce the high-frequency noises (>1 kHz) of the scanner. Before scanning, we appropriately adjusted the sound pressure level for each participant by presenting sample stimuli. The stimulus presentation and collection of behavioral data [accuracy and response times (RTs)] were controlled with the Presentation software package (Neurobehavioral Systems, Albany, CA, USA).

### MRI Data Acquisition and Analyses

For the MRI data acquisition, we used a 3.0 T MRI system with a bird-cage head coil (Signa HDxt; GE Healthcare, Milwaukee, WI, USA), and all tests were conducted in an MRI scanner. We obtained fMRI data using a gradient-echo echo-planar imaging (EPI) sequence [30 axial slices, thickness = 3 mm, slice gap = 0.5 mm, repetition time (TR) = 2 s, echo time (TE) = 30 ms, flip angle (FA) = 78°, field of view (FOV) = 192 × 192 mm^2^, in-plane resolution = 3 × 3 mm^2^]. In each run, we discarded the initial four volumes that allowed for the rise of the MR signals. We also obtained high-resolution T1-weighted images of the whole brain with a three-dimensional fast spoiled gradient recalled acquisition in the steady-state (3D FSPGR) sequence (136 axial slices, TR = 8.5 ms, TE = 2.6 ms, FA = 25°, FOV = 256 × 256 mm^2^, volume resolution = 1 × 1 × 1 mm^3^) for normalizing fMRI data.

The fMRI data were analyzed in a standard manner using SPM12 statistical parametric mapping software (Wellcome Trust Center for Neuroimaging^1^ (Friston et al., 1995), implemented on MATLAB (Math Works, Natick, MA, USA). The acquisition timing of each slice was corrected using the middle slice (the 15th slice chronologically) as a reference for the EPI data. We realigned the time-series data in multiple runs to the first volume in all runs, and further realigned the data to the mean volume of all runs. The realigned data were resliced using seventh-degree B-spline interpolation so that each voxel of each functional image matched that of the first volume.

After alignment to the AC-PC line, each participant’s T1-weighted structural image was coregistered to the mean functional image generated during realignment. The coregistered structural image was spatially normalized to the standard brain space as defined by the Montreal Neurological Institute (MNI), by using the unified segmentation algorithm with medium regularization, which is a generative model that combines tissue segmentation, bias correction, and spatial normalization in the inversion of a single unified model. After spatial normalization, the resultant deformation field was applied to the realigned functional imaging data, which was resampled every 3 mm using seventh-degree B-spline interpolation. All normalized functional images were then smoothed by using an isotropic Gaussian kernel of 9 mm full-width at half maximum. Low-frequency noise was removed by high-pass filtering at 1/128 Hz.

In the first-level analysis (i.e., the fixed-effects analysis) for each test, we set three types of conditions: reading, first listening, and second listening. Each participant’s hemodynamic responses induced by fMRI events were modeled with a boxcar function with a duration as shown in Figure 1. The boxcar function was then convolved with a hemodynamic response function. To minimize the effects of head movement, the six realignment parameters obtained from preprocessing were included as a nuisance factor in a general linear model. The images under the fMRI events and those under the baselines were then generated in the general linear model for each participant, and they were used for the “fMRI event – baseline” contrast in the second-level analysis (i.e., the random-effects analysis), which was thresholded at uncorrected *p* < 0.0001 for the voxel level, and at corrected *p* < 0.05 for the cluster level, with family-wise error (FWE) correction across the whole brain. To examine the activation of the regions in an unbiased manner, we adopted whole-brain analyses.

To calculate averaged percent signal changes for the Pre and Post sets, we focused on seven bilateral regions of interest (ROIs), which were selected from the significantly activated regions during the *second listening* events of the *Pre* sets: the inferior and middle frontal gyri (IFG/MFG), superior and middle temporal gyri (STG/MTG), inferior and superior parietal lobules (IPL/SPL), fusiform gyrus (FG), inferior and middle occipital gyri (IOG/MOG), and posterior hippocampus. The ROI of the anterior hippocampus was selected from activations during first listening events of the Post sets, and the following mask was further applied to separate *y* coordinates from the posterior part: −50 < *x* < 0, −26 < *y* < 30, −40 < *z* < 30 for the left hippocampus, and 0 < *x* < 50, −23 < *y* < 30, −40 < *z* < 30 for the right hippocampus. To separate larger clusters spanning multiple regions into each ROI, we used masks combining cortical regions defined by the Anatomical Automatic Labeling method^2^; (Tzourio-Mazoyer et al., 2002), together with the labeled data as provided by Neuromorphometrics Inc.,^3^ under academic subscription. The IFG/MFG consisted of three regions [middle frontal gyrus (Frontal_Mid_2), inferior frontal gyrus/opercular part (Frontal_Inf_Oper), and inferior frontal gyrus/triangular part (Frontal_Inf_Tri)], the STG/MTG of three regions [Heschl’s gyrus (Heschl), superior temporal gyrus (Temporal_Sup), and middle temporal gyrus (Temporal_Mid)], the IPL/SPL of four regions [superior parietal gyrus (Parietal_Sup), inferior parietal gyrus (Parietal_Inf), supramarginal gyrus (SupraMarginal), and angular gyrus (Angular)], and the IOG/MOG of six regions [calcarine fissure and surrounding cortex (Calcarine), cuneus (Cuneus), lingual gyrus (Lingual), superior occipital gyrus (Occipital_Sup), middle occipital gyrus (Occipital_Mid), and inferior occipital gyrus (Occipital_Inf)]. For each ROI, we extracted the mean percent signal changes from each participant using the MarsBaR-toolbox^4^.

## Results

Here, we present an outline of our analyses carried out on behavioral data and fMRI data. First, we assessed improvements in the reading and listening tests from Pre to Post sets in terms of the accuracy and response times (RTs). We measured RTs from the onset of choice presentation for the reading tests, and the *second* presentation of each question for the listening tests. Because listening tests were generally difficult for L2 learners, the listening stimuli were always presented twice, as in the case of original placement tests. Note that the first presentation of each question and the choices (options) was just preparatory without choosing an answer (see the “Stimuli and Tasks” section). This procedure was identical between the short and long stimuli (see Figure 1), and the RTs would become consistent if the participants could reach a correct answer by the second presentation; we thus averaged behavioral data for these stimuli together.

Next, we examined cortical activation during the presentation of Pre or Post Sets. By observing overall activation patterns for the events of reading, first listening, and second listening, we identified the most critical regions that showed significant activations, including sensory areas and language areas, as well as memory-related hippocampi. We finally conducted ROI analyses to statistically compare signal changes among various conditions: hemispheres [left, right], sets [Pre, Post], and events [reading, first listening, second listening]. We used *t*-tests for direct comparisons between individual conditions, whereas a repeated measures ANOVA (rANOVA) was used when the main effect of hemisphere or event was expected.

### Behavioral Results

**Figure 2:**
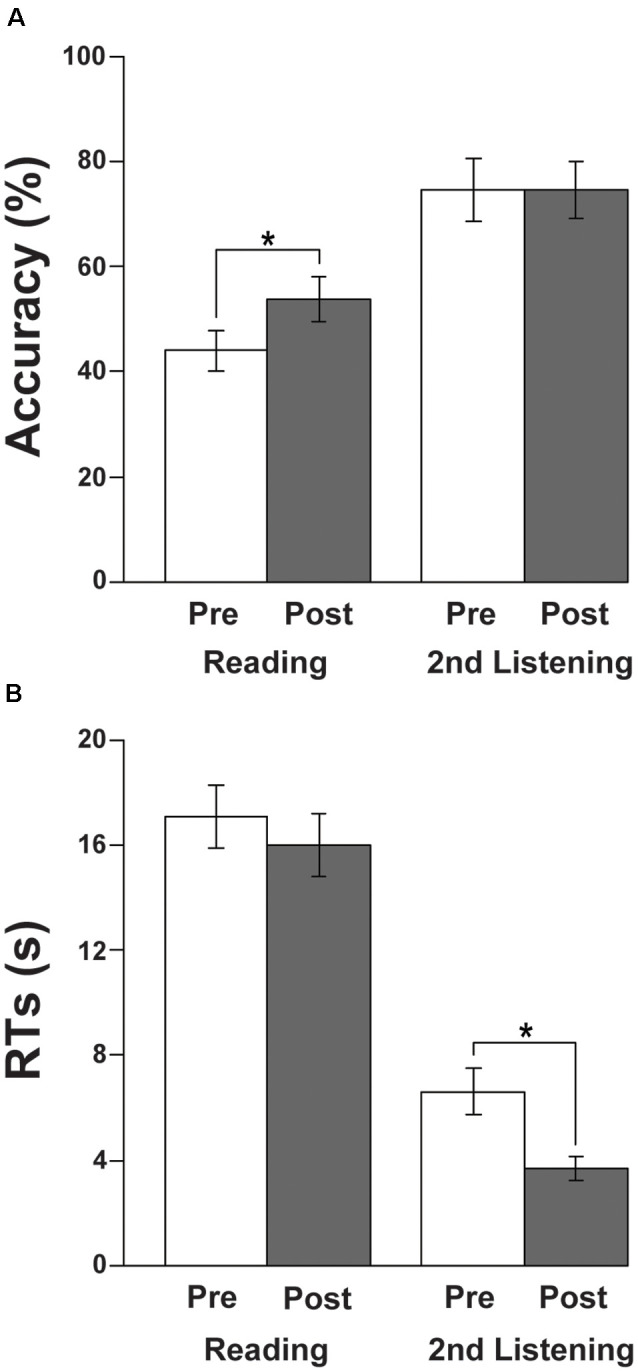
Behavioral results. **(A)** Accuracy of the reading and listening tests. Note the significant improvement in the reading tests from Pre to Post sets during the course of learning Japanese. **(B)** Response times (RTs) of the reading and listening tests. Note the significant improvement (i.e., reduction in RTs) in the listening tests from Pre to Post. The error bars denote the SEM (standard error of the mean). *p < 0.05 (paired *t*-test).

The accuracy and RTs are shown in Figure 2. With respect to the accuracy, there was a significant improvement in the reading tests from Pre to Post sets (paired *t*-test, *t*_(14)_ = 3.1, *p* = 0.0073; Figure 2A), while there was no difference in the listening tests (*t*_(14)_ = 0, *p* = 1). In regard to the RTs, we observed a significant improvement in listening tests, i.e., a reduction in RTs from Pre to Post (*t*_(14)_ = −3.1, *p* = 0.0073; Figure 2B), while there was no significant difference in the reading tests (*t*_(14)_ = −1.1, *p* = 0.29). These behavioral results indicate that there were improvements in both tests during the course of about 2 months.

Regarding the individually variable intervals between the Pre and Post sets, there was no significant correlation between the intervals and significant performance improvements from Pre to Post sets: the accuracy changes in the reading tests (*r* = 0.16, *p* = 0.6), or the RT changes in the listening tests (*r* = 0.28, *p* = 0.3).

### Overall Cortical Activation During the Presentation of Pre or Post Sets

**Figure 3:**
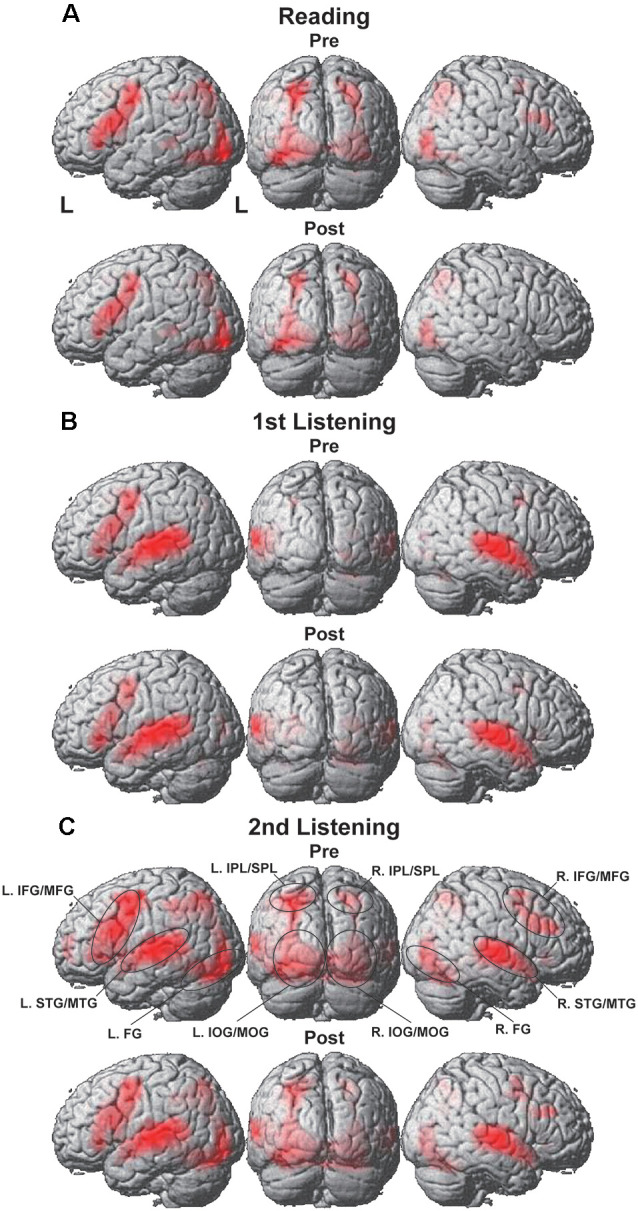
Overall cortical activation during the presentation of the Pre or Post sets. **(A)** Reading events, **(B)** first listening events, and **(C)** second listening events. Activations were projected onto the left (L) lateral, back, and right lateral surfaces of a standard brain (thresholded at uncorrected *p* < 0.0001 for the voxel level, and at corrected *p* < 0.05 for the cluster level). Regions of interest (ROIs) are denoted with black ellipses. Visual and audio areas were activated bilaterally, while the language-related frontal activations were left-dominant. IFG/MFG, the inferior and middle frontal gyri; STG/MTG, the superior and middle temporal gyri; IPL/SPL, the inferior and superior parietal lobules; FG, the fusiform gyrus; and IOG/MOG, the inferior and middle occipital gyri.

**Figure 4:**
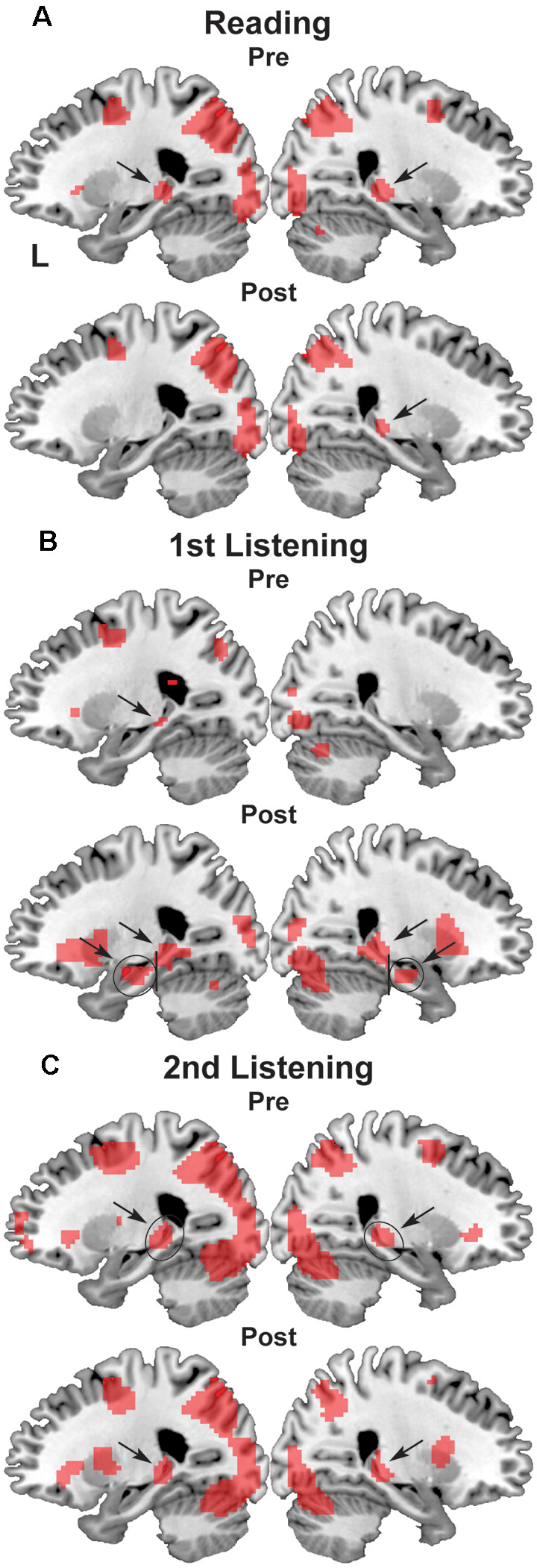
Activation in the bilateral hippocampus. **(A)** Reading events, **(B)** first listening events, and **(C)** second listening events. On the parasagittal planes (*x* = ± 24), each arrow denotes the posterior or anterior hippocampus. ROIs are denoted with black ellipses.

Significant activation was observed in both hemispheres with some notable differences for the reading, first listening, and second listening events. During the reading events (Figure 3A), prominent activation was observed in the left-dominant IFG/MFG, as well as in the bilateral IPL/SPL, FG, and IOG/MOG, i.e., the language areas and visual areas. This activation pattern was similar for the Pre and Post sets (see Figure 3 for lateral and back views, and Figure 4 for parasagittal planes). After including the individual intervals between the Pre and Post sets as a nuisance factor in a general linear model, we obtained the same activation patterns for the Post sets (Supplementary Figure 1).

During the first listening events (Figure 3B), the left-dominant activation in the IFG/MFG remained, while the visual areas were completely replaced by the bilateral STG/MTG, a part of the auditory areas. Moreover, overall cortical activation was greatly increased during the *second* listening events (Figure 3C), in which the same speech sounds were presented again together with visual stimuli (see Figure 1). In addition to the bilateral IFG/MFG and STG/MTG, visual areas of the bilateral IPL/SPL, FG, and IOG/MOG were also markedly activated during the second listening events in both the Pre and Post sets, much more than during the first listening events. These results indicate the synergistic effects of multiple modalities (i.e., vision, audition, and language), in that the sentence comprehension of auditory stimuli could be enhanced by questions and possible answers provided by visual information.

We also found significant activation in the bilateral hippocampi, which was evident on the sagittal sections of *x* = ± 24 (Figure 4). While the posterior hippocampus [MNI coordinates of local maxima: (−21, −34, −1) and (24, −28, −4)] showed consistent activation for all events (see Figure 4C), the anterior hippocampus [MNI coordinates of local maxima: (−27, −16, −16) and (24, −13, −19)] exhibited selective activation during the first listening events (see Figure 4B). This result indicates the critical role of the anterior hippocampus for the initial encoding of auditory information (see Figure 1).

### Lateralization and Changes in Activation Between Pre and Post Sets

**Figure 5:**
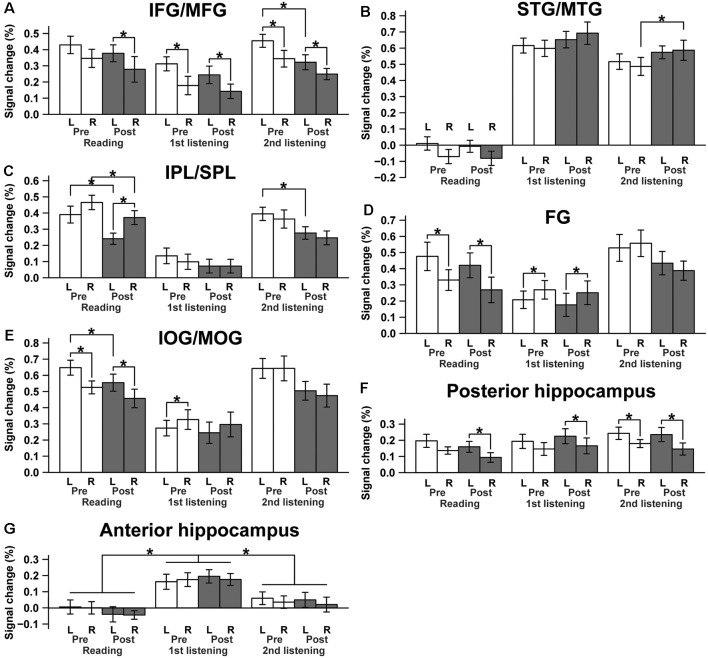
Lateralization and changes in activations from Pre to Post for each ROI. **(A)** The IFG/MFG, **(B)** the STG/MTG, **(C)** the IPL/SPL, **(D)** the FG, **(E)** the IOG/MOG, **(F)** the posterior hippocampus, and **(G)** the anterior hippocampus. *p < 0.05 (paired *t*-test).

To quantify and compare activation changes from Pre to Post for each type of event, as well as activations between ROIs of both hemispheres, we calculated mean signal changes in each ROI, separately for the Pre and Post sets (Figure 5). First, we observed a significant *reduction* in the L. IFG/MFG activations from Pre to Post during the *second listening* events (paired *t*-test; *t*_(14)_ = −2.3, *p* = 0.041; Figure 5A), though this effect was not evident in the reading or first listening events (reading: *t*_(14)_ = −0.79, *p* = 0.44; first listening: *t*_(14)_ = −0.95, *p* = 0.36). The R. IFG/MFG did not show significant changes during any of the events. Moreover, activations in the L. IFG/MFG were significantly larger than those in the R. IFG/MFG during all events except the Pre sets in the reading events. This lateralization is consistent with the critical role of the L. IFG/MFG as a grammar center. Regarding some brain regions without significant activation changes during this short period, those regions may not be critically involved in the acquisition processes, although negative results cannot exclude possible involvement.

In contrast, the R. STG/MTG showed a significant *increase* in signal changes from Pre to Post during the *second listening* events (*t*_(14)_ = 2.3, *p* = 0.038), whereas this increase was marginal during the first listening events (*t*_(14)_ = 1.8, *p* = 0.089; Figure 5B), indicating that the auditory areas became more sensitive during the course of acquiring a new language, at least at this early stage. This activation increase was in marked contrast with the results in the visual areas—namely, each side of the IPL/SPL showed a significant *decrease* in signal changes from Pre to Post during the *reading* events (L. IPL/SPL: *t*_(14)_ = −3.8, *p* < 0.005; R. IPL/SPL: *t*_(14)_ = −3.0, *p* = 0.0097; Figure 5C). The L. IOG/MOG also showed a similarly significant tendency during the reading events (*t*_(14)_ = −2.3, *p* = 0.040; Figure 5E). On the other hand, the L. FG activation was consistently stronger than the R. FG activation during the *reading* events, just like the IOG/MOG, while this tendency was reversed during the *first listening* events (Figure 5D). These results suggest the existence of differential control mechanisms of activation changes depending on the modalities and hemispheric regions.

On the other hand, the posterior and anterior hippocampi showed different activation patterns, exhibiting differences related to hemispheres or events rather than Pre/Post sets. Therefore, we used an rANOVA with three factors (hemisphere [left, right] × set [Pre, Post] × event [reading, first listening, second listening]). The posterior hippocampus showed a significant main effect of the hemisphere (*F*_(1,14)_ = 14, *p* < 0.0005) with larger activations in the left hippocampus (Figure 5F). In contrast, the anterior hippocampus showed a significant main effect of the event (*F*_(2,28)_ = 15, *p* < 0.0005) with increased activations during the *first listening* events alone, but without a hemispheric or set difference (*p* > 0.5), i.e., no significant learning effects (Figure 5G). A *post hoc* analysis further showed marked differences between the first listening events and others (reading, *t*_(14)_ = 4.9, *p* < 0.0005; second listening, *t*_(14)_ = 6.0, *p* < 0.0005). These results suggest different functional roles of the posterior and anterior hippocampi.

## Discussion

By administering a series of reading and listening tests (Figure 1) to participants who had begun to learn Japanese abroad, we obtained the following major findings. First, the left-dominant language areas, as well as bilateral visual and auditory areas, were activated (Figure 3), demonstrating the synergistic effects of multiple modalities. There was also significant activation in the bilateral hippocampi (Figure 4), indicating the expected involvement of memory-related processes. Second, consistent with the behavioral improvements from Pre to Post (Figure 2), the brain activations *decreased* in the L. IFG/MFG during the listening tests, as well as in the visual areas (the bilateral IPL/SPL and L. IOG/MOG) during the reading tests, while activations in the R. STG/MTG *increased* during the listening tests (Figure 5). These modality-dependent activation changes could not be explained by domain-general cognitive factors, such as habituation or familiarization, because we used completely different test sets for Pre and Post. Third, the posterior hippocampus showed a main effect of the hemisphere, whereas the anterior hippocampus showed a significant main effect of the event (i.e., specific to first listening events), reflecting initial encoding of auditory information alone. In summary, activation changes from Pre to Post indicate functional changes in modality-dependent networks over a short period of staying abroad, which would enable effective acquisition in an L2.

The relevance of these findings to the second language acquisition was evident from the involvement of sensory areas and language-related regions. Moreover, the decreased activations in the L. IFG/MFG are consistent with our previous hypothesis (Sakai, 2005) that such decreases reflect the saving of extraneous energy to process syntactic structures in L2. Although a further longitudinal evaluation of our participants was not possible, brain activations during the period of consolidation for the acquired linguistic knowledge would directly verify the hypothesis. The decreased activations in the visual areas may also be explained by reduced visual attention to letters and words when skills or proficiency in reading texts improved. On the other hand, the increased activations in the R. STG/MTG would be due to an improved ability to catch the sound patterns in L2, which further facilitate auditory attention to speech sounds as well. Further studies are required to clarify when activations in the bilateral STG/MTG turn to a decrease or become more focal during the subsequent development of L2 acquisition.

A previous study suggests that hemispheric asymmetry or lateralization of L2 processing depends on the similarity between L1 and L2 (D’Anselmo et al., 2013). The activation differences found in the present study might be affected by such linguistic factors, where Japanese belongs to a type of language different from the Indo-European family as L1 for the participants. Moreover, the use of a complicated writing system in Japanese (*kana*, *kanji*, and the Roman alphabet) would have required additional visual processes of identifying letters for inexperienced participants. However, our previous fMRI results of acquiring English as L2 for Japanese participants, i.e., in the reverse direction between languages from L1 to L2, have already established the basic commonality between L1 and L2 for both anatomical and functional properties in the language areas (Sakai et al., 2004, 2009; Tatsuno and Sakai, 2005; Nauchi and Sakai, 2009).

The anterior hippocampus, which is typically located at MNI coordinates close to those in the present study (22, −20, −18), has been suggested to have multiple cognitive functions: perception, imagination, and episodic memory (e.g., recall of scenes and events; Zeidman and Maguire, 2016). These proposed functional roles are consistent with the event-related activations in the present study. Indeed, perceptual *encoding* was particularly required for the initial exposure to auditory information, together with the ability to imagine what was being described in the presented speech and conversation, which were required most for the *first listening* events among the events we tested. It might be possible that increased activations during the first listening, but not during the second listening events, suggest domain-general responses to novel auditory/visual stimuli, but the complete absence of activations in the anterior hippocampus during the reading events (see Figure 5G) excluded this possibility. On the other hand, an fMRI study on associative memory showed that activations in the left posterior hippocampus were increased more during retrieval, while the left anterior hippocampus was activated more during encoding (Prince et al., 2005). The region was centered at Talairach coordinates of (−19, −41, 6), which were also close to those in the present study. Our results are consistent with this interesting double dissociation, in that the left posterior hippocampus was activated irrespective of the events we tested, but only when specific linguistic information had to be retrieved to solve the tests. This finding suggests the possible network for these subregions of the hippocampus and the modality-specific cortical regions, indicating that dynamic encoding and retrieval processes are involved in the acquisition of a new language.

## Data Availability Statement

The raw data supporting the conclusions of this article will be made available by the authors, without undue reservation.

## Ethics Statement

The studies involving human participants were reviewed and approved by the Institutional Review Board of The University of Tokyo, Komaba Campus. The patients/participants provided their written informed consent to participate in this study.

## Author Contributions

KLS and TK designed the study. TK conducted the experiment. KLS, TK, SY, and KM analyzed the data and wrote the manuscript. All authors contributed to the article and approved the submitted version.

## Conflict of Interest

The authors declare that the research was conducted in the absence of any commercial or financial relationships that could be construed as a potential conflict of interest.

## Footnotes

^1^http://www.fil.ion.ucl.ac.uk/spm/

^2^http://www.gin.cnrs.fr/AAL2/

^3^http://Neuromorphometrics.com/

^4^http://marsbar.sourceforge.net/
