# Supplementary material for: Modality-Dependent Brain Activation Changes Induced by Acquiring a Second Language Abroad
Source: Front Behav Neurosci. 2021 Mar 26;15:631957. doi: 10.3389/fnbeh.2021.631957 (PMC8032875; doi:10.3389/fnbeh.2021.631957)
Supplement: Supplementary file 1 [file Image_1.pdf]

# Modality-Dependent Brain Activation Changes Induced by Acquiring a Second Language Abroad

*Kuniyoshi L. Sakai, Tatsuro Kuwamoto, Satoma Yagi, and Kyohei Matsuya*

*Department of Basic Science, Graduate School of Arts and Sciences, The University of Tokyo, Tokyo, Japan*

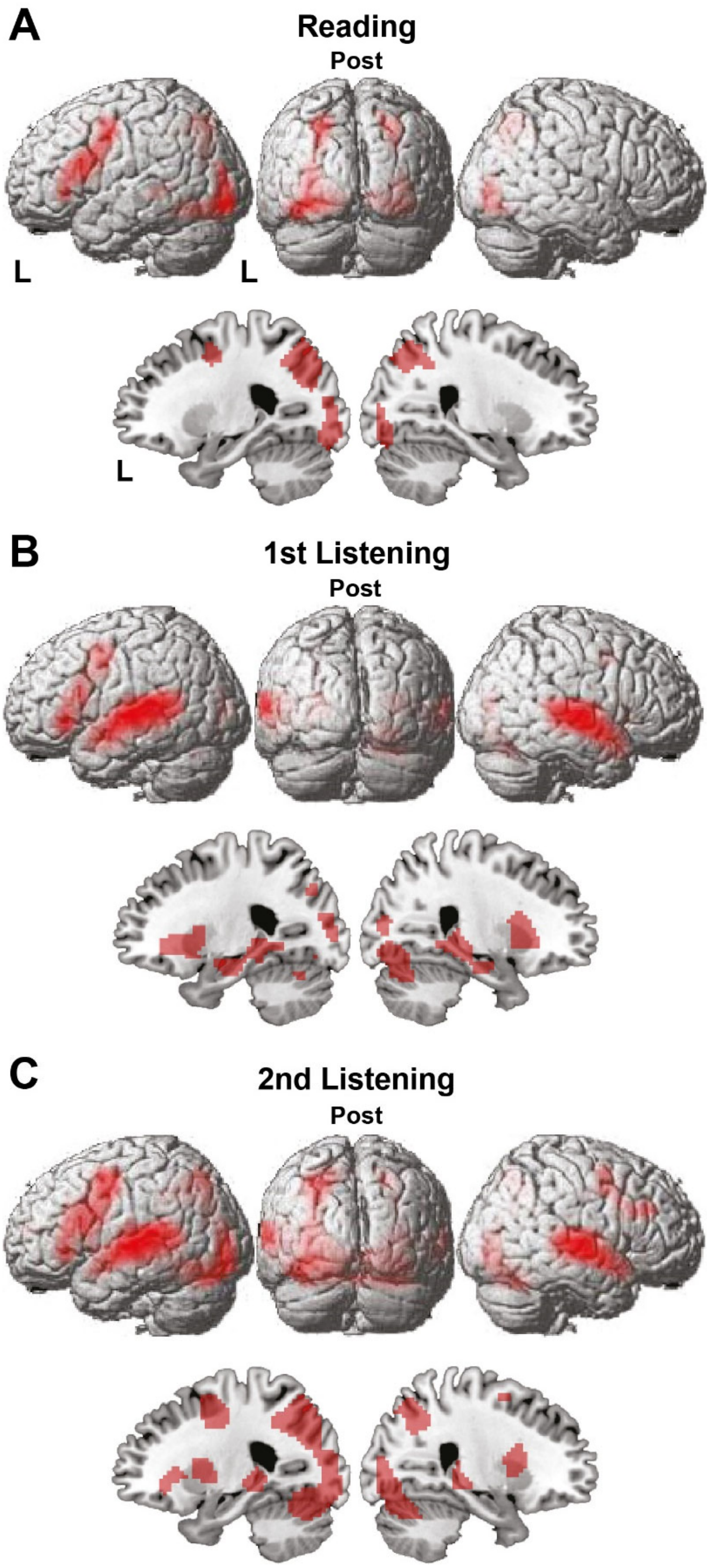


**SUPPLEMENTARY FIGURE 1** | Activation during the presentation of the Post sets, including the individual intervals between the Pre and Post sets as a nuisance factor. **(A)** Reading events, **(B)** first listening events, and **(C)** second listening events. Activations were projected onto the left (L) lateral, back, and right lateral surfaces, as well as onto the parasagittal planes ($x = \pm 24$), of a standard brain.
